# Study on quantitative diagnosis model of TCM syndromes of post-stroke depression based on combination of disease and syndrome

**DOI:** 10.1097/MD.0000000000025041

**Published:** 2021-03-26

**Authors:** Ji-Peng Yang, Hong Zhao, Yu-Zheng Du, Hong-Wen Ma, Qi Zhao, Chen Li, Yi Zhang, Bo Li, Hong-Xia Guo, Hai-Peng Ban, Hai-Ping Lin, Wen-Long Gu, Xiang-Gang Meng, Qian Song, Xiao-Xian Jin, Tao Jiang, Xin Du, Yi-Xin Dong, Hai-Lun Jiang, Nan-Fang Wu, Wei Liu, Chang Rao, Yan-Jie Tong, Yu Li, Jing-Ying Liu

**Affiliations:** a First Teaching Hospital of Tianjin University of Traditional Chinese Medicine, National Clinical Research Center for Chinese Medicine Acupuncture and Moxibustion; b Nankai University Affiliated Hospital, Tianjin; c Henan Provincial Chest Hospital, Henan; d Tianjin University of Traditional Chinese Medicine, Tianjin, China.

**Keywords:** BP artificial networks, PSD, quantitative diagnosis, stroke, TCM

## Abstract

**Background:**

Post-stroke depression (PSD) is one of the most common stroke complications with high morbidity. Researchers have done much clinical research on Traditional Chinese Medicine (TCM) treatment, but very little research on diagnosis. Based on the thought of combination of disease and syndrome, this study will establish a unified and objective quantitative diagnosis model of TCM syndromes of PSD, so as to improve the clinical diagnosis and treatment of PSD.

**Objective:**

First: To establish a unified and objective quantitative diagnosis model of TCM syndromes in PSD under different disease courses, and identify the corresponding main, secondary, and concurrent symptoms, which are based on the weighting factor of each TCM symptom. Second: To find out the relationship between different stages of PSD and TCM syndromes. Clarify the main syndrome types of PSD under different stages of disease. Reveal the evolution and progression mechanism of TCM syndromes of PSD.

**Methods and analysis:**

This is a retrospective study of PSD TCM diagnosis. Three hundred patients who were hospitalized in the First Teaching Hospital of Tianjin University of TCM with complete cases from January 2014 to January 2019 are planned to be recruited. The study will mainly collect the diagnostic information from the cases, find the related indicators of TCM and Western medicine in PSD, and clarify the relationship between different disease stages and TCM syndromes. Finally, the PSD TCM syndrome quantitative diagnosis model will be established based on the operation principle of Back Propagation (BP) artificial neural network.

**Conclusion:**

To collect sufficient medical records and establish models to speed up the process of TCM diagnosis.

## 1 Introduction

Post-stroke depression (PSD) is a kind of acquired intelligence impairment syndrome with cognitive impairment as the core symptom. It is a series of emotional disorders that occur after stroke and show symptoms other than stroke, mainly characterized by low mood, lack of interest, mental and personality abnormalities, decreased social interaction, and adaptability.^[1,2]^ According to some studies, the more severe the stroke is, the higher the incidence of PSD will be. Moreover, PSD can significantly hinder the recovery of neurological function in stroke patients and increase the mortality of stroke patients.^[3,4]^ In recent years, the incidence of PSD is increasing, ranging from about 30% to 50%. PSD is one of the most common stroke complications with high morbidity.^[5]^

The concept of entirety and treatment based on syndrome differentiation are characteristics of Traditional Chinese Medicine (TCM). “Syndrome” is the basis for TCM diagnosis of diseases, prescribing medicine and observing curative effect accordingly. Accurate syndrome differentiation is the key to achieve curative effect in treatment. Therefore, syndrome diagnosis and treatment are particularly important in TCM diagnosis and treatment of diseases.^[6]^ Due to the long course of PSD, deficiency and excess of diseases and the complex syndrome types, 4 problems have been observed in the diagnosis of PSD syndrome in TCM.^[7–10]^ First, people pay more attention to make prescription by the diagnosis of syndromes and syndrome types on the cross-section of PSD, but ignore the restriction and influence of different pathological changes and disease evolution rules of the same syndrome in different diseases. Second, people focus on the whole PSD disease but ignore the stages of the disease itself. Different TCM syndrome elements vary across PSD stages. If the whole disease is studied without stage differentiation, the mixed results could be misleading. Third, in PSD syndrome diagnosis, there are many qualitative judgments lacking unity and objectivity, which need to be solved by experts’ experience. Moreover, the former syndrome diagnosis models, without weighting factor of the main, the secondary and the concurrent symptoms of specific syndromes in TCM syndrome diagnosis, have lacked objective quantitative criteria. Fourth, the changing and evolving laws of syndromes in the process of occurrence, development and prognosis of PSD are neglected.

In summary, this study is aiming to establish a unified and objective quantitative diagnostic standard of TCM for specific syndromes in different stages of PSD, and to clarify the evolution and progression law of PSD syndromes. These achievements will play great roles in promoting the standardized research of PSD TCM syndrome diagnosis and improving clinical diagnosis and treatment.

## 2 Methods and analysis

### 2.1 Study registration

This study protocol was registered at www.chictr.org.cn (http://www.chictr.org.cn/showprojen.aspx?proj=62356) on October 19, 2020, before trial commencement (Trial registration number: ChiCTR2000039143). The patients will be selected from the medical records and fully informed of the benefits and potential disadvantages. Seeking prior consent is necessary, and they will be provided written informed consent.

### 2.2 Ethics and dissemination

This study must be conducted in accordance with the declaration of Helsinki and relevant Chinese clinical trial research standards and regulations. Before the experiment begins, the ethics committee of First Teaching Hospital of Tianjin University of TCM approved the study. The results of the trial will be reported through peer-reviewed journals and conference presentations.

### 2.3 Research type

This is a retrospective study of PSD TCM diagnosis.

### 2.4 Recruitment

This study will recruit a total of 300 PSD patients who were hospitalized patients in the First Teaching Hospital of Tianjin University of TCM with complete cases from January 2014 to January 2019. The recruitment started on 1st November 2020, and is expected to be completed on 31st October 2021.

### 2.5 Inclusion criteria

- The diagnosis is consistent with PSD;
- The age is 18 to 75 years old, both male and female;
- The information of related cases is complete;
- The course of stroke is more than 14 days.

### 2.6 Exclusion criteria

- Patients with disturbance of consciousness and obvious mental retardation and aphasia;
- Patients with positive personal and family history of mental disorders;
- Patients with serious heart disease, heart failure, liver dysfunction, renal insufficiency, respiratory failure, malignant tumor, gastrointestinal bleeding, etc at the onset of the disease, they are not expected to complete the long-term follow-up;
- Pregnant and lactating women.

### 2.7 Blind method

Data collection and recording will be done by Clinical Research Coordinator (CRC). The data statistics will be completed by a third party statistician.

### 2.8 Main indicators

- TCM syndrome of stroke and PSD;
- Four diagnostic methods information of TCM Inspection: tongue appearance, complexion, etc; Smelling: smelling halitosis and other abnormal body odor; Inquiring: sweating and defecation; Palpating: pulse condition, whether to touch gall, tumor, scrofula, and swelling;
- Original prescription of TCM;
- Score of TCM syndrome score scale;
- Stage of PSD.

### 2.9 Secondary indicators

- Demographic data: age, gender, height, weight, etc;
- Influencing factors of disease diagnosis: past history and combined medication;
- General physical examination;
- Laboratory examination;
- Imageological examination.

### 2.10 Data collection

Researchers who collect patient information during the study will receive rigorous training. The date is collected and conducted in accordance with relevant regulations and standard operating procedures. The borrowed paper-based medical records and related materials will be stored and locked in dedicated area of hospital. The collected electronic information will be stored in dedicated computer or hard drive with password protection. The experimental information can be secured not to be exposed. The host of the project will be fully responsible for the confidentiality of the medical records.

### 2.11 Sample size estimation

Since Back Propagation (BP) artificial neural network is the core method for the construction of syndrome model in this study, this method needs a large amount of data support in the parameter fitting process, and there is no exact method to calculate the sample size at present. According to some relevant studies,^[11–14]^ it is suggested that sample size should be not less than 200. Combined with the admission status of PSD in this hospital and 20% verification data, this study plans to collect 300 cases.

### 2.12 Statistical methods

Demographic data, general physical examination, laboratory examination, imageological examination, 4 diagnostic methods information of TCM Inspection, and original prescription of TCM are input by SPSS17.0 software. All data information is converted into numbers, and there is no text information except the variable names. The coding principle of grade data is the occurrence probability of each variable grade. The assignment principle of qualitative variables is 1 for existence and 0 for nonexistence.

**Figure 1:**
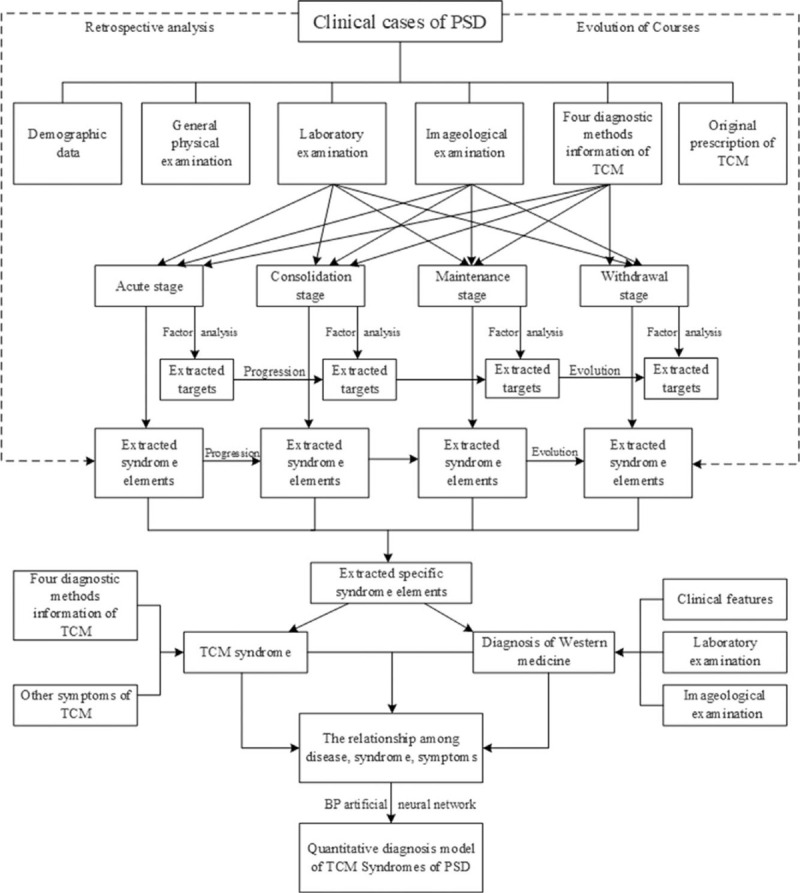
Study flow.

The TCM diagnostic information in the clinical data is included in the analysis. Factor analysis is used to extract the syndrome elements and targets of PSD in different stages (acute stage, consolidation stage, maintenance stage, and withdrawal stage). Principal component analysis is performed for each group. According to the values of gravel map and characteristic root, the significant common factors are extracted. According to the symptom indexes of common factors, the syndrome elements are classified and their targets are defined. According to the PSD associated indexes of traditional Chinese and western medicine combining with disease and syndrome, the main PSD syndrome types and the main TCM symptoms under specific syndromes are determined. Based on BP artificial neural network, the quantitative diagnosis model of TCM syndromes in PSD will be established (Fig. 1).

### 2.13 Case shedding and termination

- *Case shedding*: If serious adverse events occur, the test subjects will be stopped according to the doctor's judgment. For whatever reason, the patient is unable to continue the clinical trial, the trial will be terminated.
- *Termination criteria*: If serious adverse events occur, the test subjects will be stopped according to the doctor's judgment.
- *Treatment*: Contact the subjects or their family members in time to ask the reason, complete the evaluation items that can be completed, and properly keep the relevant test data.

### 2.14 Quality assurance and quality control

Subject group members are fixed to 3 to 5 people, who are asked to take unified training in order to familiar and master the experimental implementation program and carry out the study in strict accordance with the requirements of the scheme. One to two people are assigned by the hospital to frequently monitor the research situation, and check the medical records in the early, middle, and late stages during the experiment, and solve potential issues in timely manner.

### 2.15 Patient and public involvement

No patients and the public involved.

## 3 Discussions

Every disease has its own internal rules of occurrence and development that are different from other diseases. The same syndrome of TCM could be affected by different pathological mechanisms in different diseases, and each kind of disease has its own course stage.^[15]^ Therefore, the combination of disease and syndrome is of great significance for the establishment of standard quantitative diagnostic criteria for PSD, and opens an avenue for development of TCM. The establishment of a quantitative diagnosis model of TCM syndromes based on the combination of disease and syndrome can make the diagnosis of TCM syndromes more objective, and provide a basis for the clinical treatment and efficacy discrimination of diseases.^[16]^ Quantitative diagnosis of TCM syndromes mainly summarizes the laws between elements and syndromes through mathematical analysis methods, before the mathematical model of syndrome diagnosis is established.^[8]^

At present, the commonly used syndrome modeling methods are divided into linear modeling method and nonlinear modeling method.^[17,18]^ However, TCM syndrome system is characterized by nonlinear complexity, and there are a large number of multicollinearity and cooperative relationships among symptoms. Although linear modeling method is conducive to the mathematical description of complex problems, it is difficult to accurately simulate the complex relationship between symptoms and syndromes, and it is even more difficult to approximate the real appearance of TCM syndromes.^[19]^ Nonlinear modeling method is suitable for TCM system modeling.

Artificial neural network has become the main modeling method for nonlinear system discrimination because of its strong nonlinear mapping ability.^[20]^ BP artificial neural network is multi-layer forward neural network which is based on error Back Propagation algorithm.^[21,22]^ With capability of arbitrary nonlinear mapping between the input and the output, BP artificial neural network has been widely used in function approximation, pattern recognition, data compression and other fields. This method has also been demonstrated to perform nonlinear modeling of TCM syndromes.^[23,24]^ This study will continue efforts to implement it in the quantitative diagnosis of TCM syndromes in PSD in order to standardize the syndrome classification of PSD.

### 3.1 Limitations

(1) The TCM diagnostic information of many medical cases is incomplete and difficult to complete because of the retrospective study, which leads to the exclusion of the study.
(2) Patients of the study are mainly concentrated in the same region, and the research output may not be applicable in other regions.

## 4 Conclusion

This study was planned to collect sufficient medical records to make the results more scientific and accurate, and the establishment of the model will accelerate TCM diagnosis, which is less influenced by subjective factors. The results of this study may be useful to clinicians, practice guide developers, researchers, decision makers, and so on.

## Author contributions

JPY and HZ contributed equally.

JPY, HZ, JYL designed this study.

JPY, JYL, WLG, XGM, QS, HPB, HPL, HLJ, and YXD provided statistical expertise and supported the development of the statistical analysis plan.

JPY, NFW, WL, YL, CR, and YJT will recruit patients and extract data.

JPY, HXG, HPB, XXJ, and TJ will analyze data. All authors approved the version to be published.

**Conceptualization:** Jipeng Yang, Hong Zhao, Jingying Liu.

**Data curation:** Jipeng Yang, Hongxia Guo, Haipeng Ban, Haiping Lin, Wenlong Gu, Xianggang Meng, Qian Song, Xiaoxian Jin, Tao Jiang, Xin Du, Yixin Dong, Hailun Jiang, Nanfang Wu, Wei Liu, Chang Rao, Yanjie Tong, Yu Li.

**Formal analysis:** Jipeng Yang, Hongxia Guo, Haipeng Ban, Xiaoxian Jin, Tao Jiang.

**Investigation:** Jipeng Yang.

**Project administration:** Jingying Liu.

**Software:** Jipeng Yang, Haipeng Ban, Haiping Lin, Xianggang Meng, Qian Song, Xiaoxian Jin, Tao Jiang, Xin Du, Yixin Dong, Hailun Jiang, Nanfang Wu, Wei Liu, Chang Rao, Yanjie Tong, Yu Li.

**Writing – original draft:** Jipeng Yang, Yuzheng Du, Hongwen Ma, Qi Zhao, Chen Li, Yi Zhang, Bo Li, Jingying Liu.
